# In Reply: Incorporating teleoncology practices in the undergraduate medical curriculum

**DOI:** 10.2217/fon-2020-0985

**Published:** 2020-11-27

**Authors:** Ronaldo Elkaddoum, Fady Gh Haddad, Roland Eid, Hampig Raphael Kourie

**Affiliations:** ^1^Department of Hematology-Oncology, Faculty of Medicine, Saint Joseph University of Beirut, Riad El Solh, 11-5076, Lebanon

**Keywords:** COVID-19, medical curricula, medical students, telemedicine, teleoncology

To the Editor,

We were very happy to read the Letter to the Editor in response to our article ‘Telemedicine for cancer patients during COVID-19 pandemic: between threats and opportunities’ [1]. The letter highlights a major aspect of oncology – oncological education for medical students. Although this topic has been overlooked for a long time, teleoncology could indeed be a great way to introduce medical students to oncological care.

Under-represented in medical curricula, clinical oncology is one field where medical students doubt their capacities [2]. To tackle the issue, undergraduate students should have early contact with oncological patients with whom they will learn to communicate [3]. Since these patients are usually immunocompromised, the flow of visitors or personnel to their room should be monitored. With COVID-19, stricter distancing measures should be taken since cancer patients are at a higher risk of complications and more serious events than the general population [4]. Between the necessity of a practical teaching in oncology for medical students, and the obligation of avoiding the unnecessary exposure of oncological patients to COVID-19, the dilemma remains. Considering that the ideal solution would be to allow students to directly communicate and assess patients, teleoncology – the use of various technologies in cancer care – presents itself as a solution for clinical learning in times of pandemics. Technically, there could be many ways to introduce students to video consults. The first way is recording the consult session after a clear written consent by the patient. Although in this way students will not be able to directly contact the patients, they could benefit from a detailed narration of the consult and points they should watch. The second way is the direct connection of the students to the consult chat. One major point here is that everyone in the room should be presented and, of course, the patient should consent on it. This form of participation allows medical students not only to watch but also to participate when given the permission in assessing the patients, explaining some points to them and answering their questions, all under the direct supervision of the attending practitioner.

Prior to any participation in such meetings, students should get introduced to telemedicine and how to use it in the most efficient way. In another article [5] published by our team, we discussed how oncologists can educe the psychological difficulties of cancer patients by implementing some changes in the settings of their video consult. The following points are also applicable for participating medical students. First of all, clinicians should adopt a patient-centered approach, where patients feel their individuality in the treatment plan. Another improvement could be the administration of an emotion or distress thermometer, which allows the oncologists or the trainees to pay attention to their patients’ distress. Finally, some improvements can be done during the consultation, such as maintaining a stable internet connection, adjusting the gazing angle to make eye contact and conveying empathy by adapting the tone of the conversation to the state of the patient.

Another opportunity could be participation in multidisciplinary teams’ meetings. This is where senior physicians can play a crucial role in introducing their students to decision making in oncology by asking them for input and giving them time to ask questions. Another way to involve students is to give them the opportunity, when needed, to do a daily follow-up of patients’ physical activity, distress and new symptoms.

Since the transition to teleoncology was unprepared, it is normal that medical schools were not ready to translate observational internships to an online setting on the spot. Nevertheless, after many months since this shift, the time has come to train medical students on the protocol of online consults and emphasize the benefits of their active participation. The early implementation of teleoncology internship can also be beneficial in sharing knowledge from overseas and establishing exchange programs or international summer schools in oncology to resume a highly appreciated [6] part of medical studies. Students, who are usually more active than their professors on social media, can also share evidence-based knowledge, especially in the preventive share of oncology [7].

In conclusion, teleoncology seems to offer huge room for improvement in oncology education for undergraduate medical students. Medical schools are invited to open this new door for their students to upgrade oncological training, especially considering that the students of today will be the oncologists of tomorrow.
